# Editorial Department

**Published:** 1889-04

**Authors:** 


### EDITORIAL DEPARTMENT.

### REVIEW OF AN ARTICLE OF WILCKENS ON THE TRANSMISSIBILITY OF COLOR IN HORSES.

BY R. BARON, PROFESSOR OF ZOOTECHNICS IN RECUEIL DE MEDICINE VETERINAIRE.

The author first studies the results in thoroughbreds.

The sire and dam of the same color or of different colors.

In the first case, that is in uniting those of the same color, the transmission was perfect 856 times in a thousand of the progeny.

In the second case, the stallion transmitted his coat 437 times and the mare 508 times—a total of 945 cases of unilateral transmission in a thousand.

There remains fifty-five cases of mixed, combined or odd coats.

The color bay was transmitted 529 times unilaterally by the male and 615 times by the female in a thousand.

The color black occurred much less frequently. There were only 116 cases transmitted by the stallion and ninty-two by the mare in a thousand crosses.

In half-blood animals, in a thousand couplings between animals with markings, the progeny have shown markings 873 times.

Outside of the markings there were 367 foals with the paternal coat and 555 with the maternal.

Seventy-eight others were more or less polychromatic.

In coupling similars of a sorrel color the enormous result of 946 in a thousand resulted.

In coupling dissimilars, a marked tendency for bay to appear resulted; 554 times for the stallion, 708 times for the mare.

Again, as in the previous case, black showed but a feeble tendency to be reproduced; 132 times from the sire, and 210 times from the mare, each in a thousand cases.

### PURE AND HALF-BRED ARABS.

The sire and the dam being of the same color, it was transmitted 837 times in a thousand.

The sire and the dam of different colors, the former transmitted his color 313 times in a thousand, and the dam's color was given in 566 cases.

The remaining 121 cases were of various colors.

The color gray, from similars, was reproduced 900 times in a thousand.

If the dam only was gray, it was still transmitted 729 times in thousand.

Bay in the dam was transmitted 551 times in a thousand.

Black was again rarely reproduced ; 125 cases from the sire and 190 cases from the dam.

The appearance of a color totally different from that of the immediate progenitors is classically attributed to ancestral heredity. It may be noted that in these cases the color is almost invariably sorrel.

The transmission appears independent of the sex of the product. At the same time dark bay is found 1091 times in the filly for a 1000 times in the colt, especially in crosses.

In cases where the coupling of the same color in gray has been carried out regularly, there have been less colts than fillies of the same color.

The contrary resulted in the coupling of disimilar colors ; 1000 colts for 948 fillies.

But for the sorrel, the coupling of dissimilars did not produce an analagous effect. For each 1000 sorrel colts there were 1013 fillies of the same color.

The same law applies for the black colt ; for each 1000 black colts, there were 1036 black fillies.

It is probable, from comparing the statistics that mares have a peculiar power of transmission when it is a question of the color bay.

REFLECTIONS.

Wilcken's is one of the most justly esteemed Zootechnicians ; and if we comment on his work it is to praise it in the greatest degree. It is evident that the well collected statistics, based on 50,000 horses, ought to establish in a very satisfactory manner the laws of hereditary transmission in general and those of the color in particular. Still there seems to be a point of scientific method, not even sufficiently proved even by Wilckens.

It often happens that statisticians are "cerebral monsters" in that which concerns the psychology of proof. It is sufficient, for example, to have a little knowledge of the questions of heredity, to understand the quasi-impossibility of establishing, not the transmission but the non-transmission of certain characters. To give an appearance of strictness and ease to researches, heredity in general has been confounded with heredity strictly continuous and immediate. It is an error.

Again, I acknowledge I do not recognize the "law of similars" in the imperfect applications announced above. The coupling of two reproductors provided with the same anatomo-physiological characters leads to the probability of transmission only when each of the reproductors comes in its turn from similar parents and so on, back to the origin of the type considered.

In the third place. There exists without doubt in the group of caballines, a certain number of definite coats, which always reappear, regularly or irregularly.

But to leave this *postulatum* a little vague, if we wish to be precise, we lose ourselves very fast as no one is yet able to define the number and to specify the harmonious primary colors.

I believe, personally, that there are "sexual liveries" analagous to the differential characters of the Malay butterfly and other polymorphous arthropodes. H.

---

BRAIN OF PARRAKEET.—Austin F. Park, who is engaged in a series of investigations on the weight of the brain, eyes and other organs in birds, writes us the following concerning a Parakeet obtained from the Central Park Menagerie, as stated in his notes :—

No. 881 ; *Conurus jendaya ;* somewhat thin in flesh ; diameter of iris, 5.4 in. ; weight of gross bird, 1395 grs. ; Contents of stomach, 8 grs. ; net bird, 1387 grs. ; 2 eyes, 20 grs. ; Brain, 67 grs. ; cerebrum, 52.9 grs. ; optic lobes and medulla, 10.1 grs. ; cerebellum, 4 grs.

| | | | |
|---|---|---|---|
| Ratio of bird to brain, | 20.70 | Ratio of brain to cerebrum, | 1.25 |
| Ratio of bird to cerebrum, | 26.22 | Ratio of brain to optic lobes and medulla, | 6.63 |
| Ratio of bird to 2 eyes, | 69.35 | Ratio of brain to cerebellum, | 16.75 |
| Ratio of cerebrum to cerebellum, | 13.22 | | |

| | | | |
|---|---|---|---|
| Percentages in bird : | | Percentages in brain : | |
| Brain, . . . . | 4.83 | Cerebrum, . . . . | 78.96 |
| 2 Eyes, . . . . . | 1.44 | Optic lobes and medulla, | 15.07 |
| Bird (2 eyes and brain), | 93.73 | Cerebellum, . . . | 5.97 |
| Bird, . . . | 100.00 | Brain, . . . | 100.00 |

The percentage of the cerebrum in the brain of this Parakeet is nearly as great as in the brain of any bird that I have examined. The relative proportions of the cerebrum, cerebellum, and optic lobes and medulla, in the brain of this bird, are nearly similar to those in the brains of some Owls and Woodpeckers examined by me. The *ratio of the brain as a whole in this Parrakeet* is larger than in any Owl or Woodpecker that I have noted *in like fleshy condition.*

---

A Horn on the Margin of a Goat's Ear.—Dr. J. H. Steel published this case in the "Journal of the Bombay Natural History Society." The peculiarity affected the posterior margin and both surfaces of the right ear at about the middle third of the margin three inches from tip. On the outer surface a semicircle of the skin about half an inch in diameter had undergone warty change, forming an irregular horny mass, the area of which was extended by the circular base of the horn, which grows from the inner surface. It measured five inches in length and one-and-one-half inches in diameter. After noting the different kinds of these irregularities—Atavisms, Degenerate Organs, etc.—the doctor concluded the case to be one of Keratoma, or horn tumor, and referred to a paper by Dr. Bland Sutton published some time ago in this journal. C.

---

The Bressa Prize.—The Royal Academy of Sciences of Turin, gives notice that the term for competition of the seventh Bressa Prize has begun, to which, according to the testator's will, scientific men and inventors of all nations will be admitted. A prize of 12,000 lire ($2,316) will therefore be given to the scientific author or inventor, whatever be his nationality, who during the years 1887–90 "according to the judgment of the Royal Academy of Sciences of Turin, shall have made the most important and useful discovery, or published the most valuable work on physical and experimental Science, Natural History, Mathematics, Chemistry, Physiology and Pathology, as well as Geology, History, Geography and Statistics." The term will be closed at the end of December, 1890. The prize will in no case be given to any of the National Members of the Academy of Turin, resident or non resident.

---

The Military Horse Supply.—In the event of a war in Europe, Germany would require 400,000 horses; France, 375,000; Austria, 200,000; and Russia, 400,000. Should there be a war, therefore, the Powers would require 1,375,000 horses to begin with, and more from time to time as the animals should be killed or used up in service. With the United States as a coming great horse market, the Government has a duty to perform. Our breeders must be guided and stimulated by proper reward to use greater care in the animals they produce.

---

ACCORDING to Dr. J. B. Sutton, animals are not free from certain diseases thought to be referable in man to his erect position. One-fourth of the female monkeys dying in the London Zoological Gardens have displacement of the uterus, and the same disease occurs in the lioness, tapir, cape hunting dog, pigmy hog, antelope, etc., and in domesticated mammals. Two cases of inguinal hernia in monkeys are recorded, and the disease is said to be common in horses.

---

A part of the copy of Dr. Bodamer's article on Actinomycosis having been mislaid, the remainder will appear in the July journal.

